# High-Efficiency Sub-100 nm Gate GaN HEMTs Enabled by Two-Step SiN_x_ Layer Etching Technique

**DOI:** 10.3390/mi17080889

**Published:** 2026-07-25

**Authors:** Xiangxin Cao, Pengfei Wang, Minhan Mi, Xinyu Liu, Dongxu Xiao, Xiang Du, Maoteng Lu, Feiyang Chen, Zhentong Huang, Jingyun Yao, Yinyin Liang, Kehui Cui, Xiaohua Ma, Yue Hao

**Affiliations:** State Key Discipline Laboratory of Wide Band-Gap Semiconductor Technology, School of Microelectronics, Xidian University, Xi’an 710071, China; 19991244708@163.com (X.C.); pfeiwang1995@163.com (P.W.); 25251215492@stu.xidian.edu.cn (X.L.); xdx1835933002@163.com (D.X.); duxiang@stu.xidian.edu.cn (X.D.); 23111213485@stu.xidian.edu.cn (M.L.); asdrgh_ok@163.com (F.C.); 25251111640@stu.xidian.edu.cn (Z.H.); jyyao02@163.com (J.Y.); yinyinliang02@outlook.com (Y.L.); 22009100658@stu.xidian.edu.cn (K.C.);

**Keywords:** GaN HEMT, millimeter-wave, scaled gate-length, Ar-free etching, high-efficiency

## Abstract

This paper proposed a high-efficiency thin-barrier gallium nitride (GaN) high electron mobility transistor (HEMT) with scaled gate-length (*L*_g_) for millimeter-wave (mmW) frequency applications. A two-step etching process is adopted in this work, where an Ar-free etching was used for the secondary etching (SE) step. After partial etching of the Silicon nitride (SiN) passivation layer in the gate foot region, the *L*_g_ can be stably controlled at <100 nm, the root mean square (RMS) roughness of the AlGaN barrier layer is 0.248 nm, and the mobility degradation has been effectively suppressed. The fabricated device achieved a maximum drain current density (*I*_DS.max_) of 1644.2 mA/mm, a peak transconductance (*g*_m.max_) of 764.1 mS/mm, and a current collapse ratio of 4.7%. In addition, the current gain cutoff frequency (*f*_T_) of 105.4 GHz and maximum oscillation frequency (*f*_MAX_) of 154.4 GHz were obtained at drain source voltage (*V*_ds_) of 8 V. Furthermore, at 30 GHz, the fabricated device achieved a saturated output power density (*P*_sat_) of 4.4 W/mm and a maximum power-added efficiency (*PAE*_max_) of 66.0%, with a significant improvement of 15.7% and 5.3% compared with the device fabricated by Ar-containing etching, demonstrating the superiority of the Ar-free etching approach.

## 1. Introduction

Owing to its high two-dimensional electron gas (2DEG) concentration, high electron saturation velocity, and high breakdown electric field, gallium nitride (GaN) high electron mobility transistor (HEMT) has been widely applied in the field of radio frequency (RF) power amplifier (PA) [1]. Meanwhile, RF systems are advancing toward millimeter-wave (mmW) frequency bands, which serve as a key technological driving source in numerous emerging communication fields, particularly in 5G mobile communication [2]. To accommodate higher application frequency bands, the barrier thickness and gate-length (*L*_g_) of GaN HEMTs are continuously scaling down [3]. This requires more precise control of the etching process during the scaled gate fabrication process.

Silicon nitride (SiN) is a widely adopted passivation layer to prevent current collapse [4]. Partial etching of SiN typically induces etching damage in the vicinity of the gate region [5,6]. This increases the surface roughness of the barrier layer. As a result, the 2DEG experiences scattering, which reduces electron mobility [7], even causing elevated leakage current and degraded device efficiency [8]. For thin-barrier devices, the distribution of the 2DEG is closer to the device surface, making it more susceptible to scattering effects. Therefore, precise control of the etching depth is required to ensure the complete removal of the SiN layer in the gate foot region while minimizing the surface damage to the barrier layer during the etching process [9,10].

For RF devices, the lateral expansion (*L*_E_) during etching limits *L*_g_ scaling. This increases carrier transit time and thereby reduces the maximum stable gain (*MSG*) and power-added efficiency (*PAE*) of the device [11]. The *L*_E_ originates from two sources: first, etching is not perfectly anisotropic, which results in lateral etching during the removal of SiN. Second, the F-based etching plasma also attacks the photoresist (PR), causing the continuous lateral widening of the PR trench during the etching process. Conventional SiN etching techniques are mature and widely used in industry. Prior studies have reported the influence of its etching parameters on the etching process [12,13,14,15]. Ar plasma has been proven to enhance etching anisotropy [16], but it usually introduces more surface damage [17,18]. Low-damage etching techniques, such as digital etching [19] and soft-landing etching [20], have already been reported in previous studies. However, they are focused on mitigating the damage of conventional etching, and it is difficult to achieve sub-100 nm trenches.

Therefore, this work explored the mechanisms of the two-step etching approach and developed a corresponding process to achieve sub-100 nm *L*_g_. An Ar-free secondary etching process was adopted to reduce the etching damage. The root mean square (RMS) roughness decreased to 1/10 of that obtained with Ar etching, and the mobility was also significantly improved. In addition, a comprehensive analysis of the fabricated devices revealed that the Ar-free etching process significantly enhances overall device performance, achieving a saturated output power density (*P*_sat_) of 4.4 W/mm and a *PAE*_max_ of 66%. It demonstrates that the Ar-free etching is a superior low-damage etching scheme, particularly well-suited for thin barrier layers.

## 2. Experiment

### 2.1. Two-Step Etching Principle

For the F-based etching process in this work, the dissociation and ionization of carbon tetrafluoride (CF_4_) generate F radicals, which react with SiN to form volatile gases. The process is as follows [21]:

e^−^ + CF_4_ = CF_3_ + F + e^−^ (1)

e^−^ + CF_4_ = CF_3_^+^ + F + 2e^−^ (2)

Si_3_N_4_(s) + 12F(g) = 3SiF_4_(g) + 2N_2_(g) (3)

Here, CF_3_^+^ is a charged ion with a physical bombardment effect, F is an active radical with strong chemical reactivity, and SiF_4_ is an extremely volatile gas produced by the reaction, which is discharged from the reaction chamber through the vacuum system and exhaust gas treatment device.

**Figure 1:**
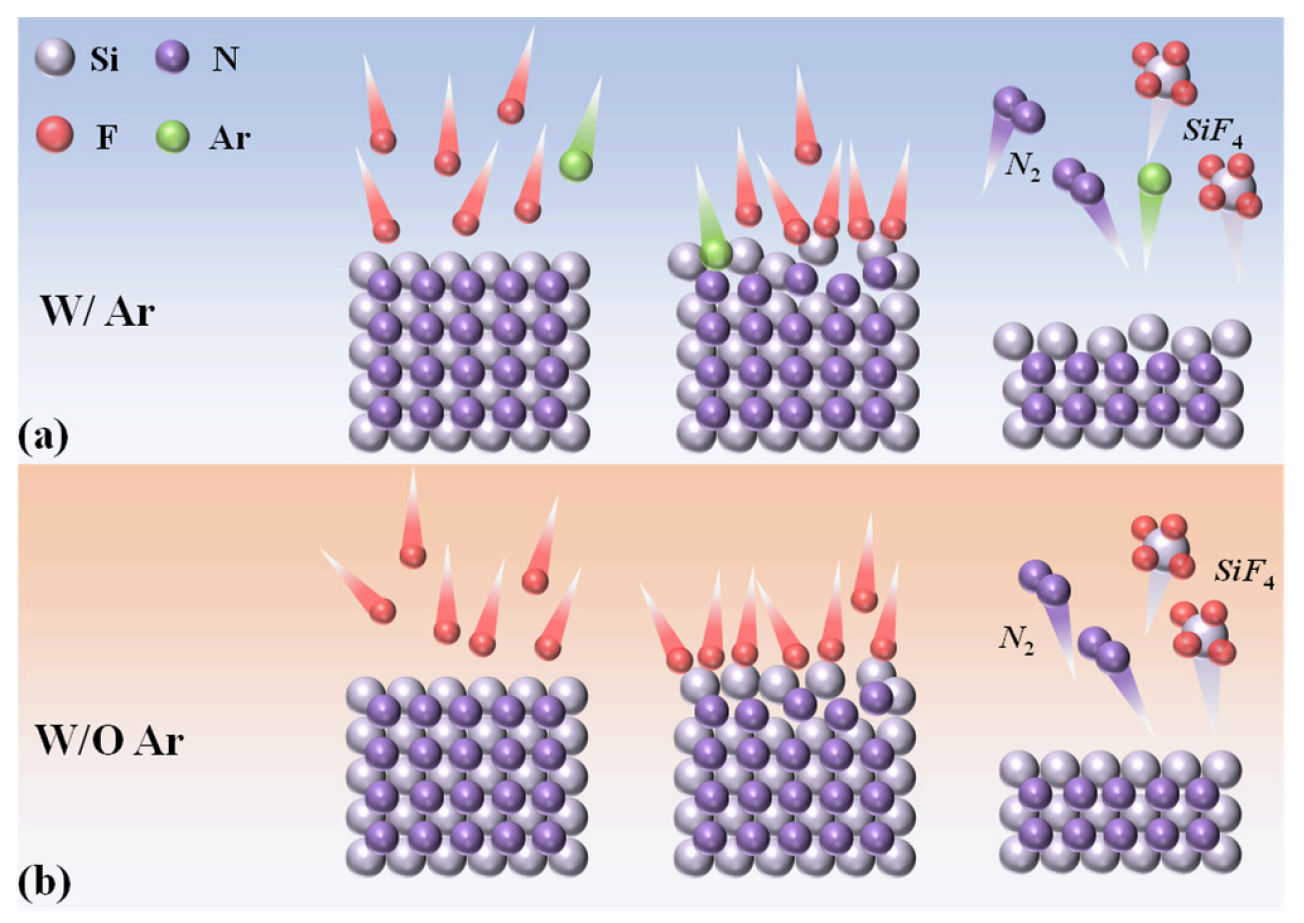
The principle of the etching scheme (**a**) with and (**b**) without Ar during the SE process.

Scaling *L*_E_ usually requires increasing the RF power to impart a higher longitudinal velocity to the etching gas. The addition of Ar to the etching gas can also enhance the etching rate and anisotropy [16]. Figure 1a,b shows the effects of the presence or absence of Ar during the etching process. However, these operations will exacerbate etching damage, so single-step etching struggles to balance low etching damage and limited *L*_E_.

Therefore, a two-step etching process consisting of primary etching (PE) and secondary etching (SE) is necessary. The PE step has a high etching rate and minimal *L*_E_, which rapidly removes most of the passivation layer and also determines the profile of the gate trench [22]. For the SE step, the etching power and the flow rate of reactive gases are reduced. This slows down the etching rate. Therefore, it minimizes damage to the barrier layer as much as possible while removing the remaining SiN.

### 2.2. Comparison of Two Etching Methods

Based on the advantages of the two-step etching process, the passivation layer etching scheme in this work consists of two parts: the PE process and the SE process. For the PE process, the etching gases are CF_4_ and Ar, with a flow rate ratio of 80/20 standard cubic centimeters per minute (sccm). The Inductively Coupled Plasma (ICP) power is set to 1000 watts (W), and the RF power is set to 150 W. Its etching rate is 7.69 nm/s for sub-100 nm trenches. The SE process includes two schemes, namely WA-SE (with Ar) and WOA-SE (without Ar). For the WA-SE, the etching gases remain CF_4_ and Ar, with a flow rate of 40/10 sccm. The ICP and RF powers are set to 250 W and 40 W, respectively. The etching rate decreased to 0.56 nm/s due to the reduction of the etching gas flow rate and plasma power. For the WOA-SE scheme, the etching gas is only CF_4_, with a flow rate of 50 sccm (maintaining the same total gas flow rate as in WA-SE). The ICP power and RF power are consistent with WA-SE, but because of the absence of Ar, the etch rate is only 0.51 nm/s.

**Figure 2:**
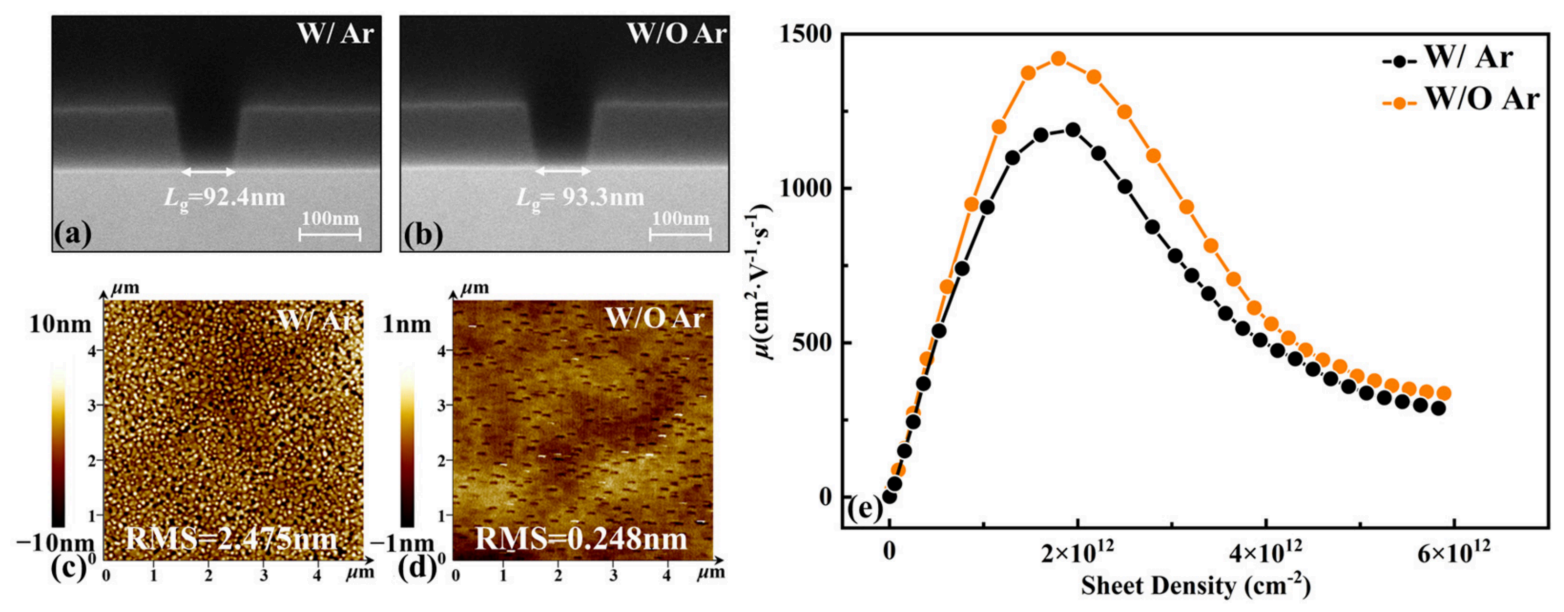
FIB images of trenches etched by (**a**) WA-SE and (**b**) WOA-SE. AFM images of trenches etched by (**c**) WA-SE and (**d**) WOA-SE. (**e**) Field-effect mobility of two schemes.

Two different etching methods are adopted in this work: PE + WA-SE and PE + WOA-SE. For device fabrication, the total etching depth of the SiN passivation layer is fixed at 120 nm. Tailored etching durations were set to achieve a 100 nm etching depth of the SiN passivation layer in the PE step, while the SE step only removes the remaining 20 nm of the SiN layer. Under the identical total etching depth condition, we quantitatively compare the surface damage induced by these two-step etching methods. As shown in Figure 2a,b, the cross-sectional morphology of the etched trenches was characterized by focused ion beam (FIB) measurements. Their sidewalls are all close to vertical. Meanwhile, it can be seen that the *L*_g_ obtained by the two etching schemes is basically consistent, with both below 100 nm. This result indicates that only removing Ar in the SE process has little impact on the *L*_g_ and the trench profile. The etching depth ratio of PE to SE is 5:1 during device fabrication. With a low etching rate and a small proportion of total etching depth, the SE step exerts a minor effect on the trench profile. The PE process exhibits excellent anisotropy, resulting in minimal *L*_E_, which is beneficial for achieving scaled *L*_g_.

The reason is attributed to the sufficient gas flow and high operating power in the PE process. Elevated ICP power increases the reactive ion concentration, enhancing the etching rate. High RF power improves the energy of ions bombarding the SiN layer, increasing etching verticality and etching rate, too [23].

The surface morphologies of trenches were analyzed using atomic force microscopy (AFM) to measure RMS roughness across a 5 µm × 5 µm area. Due to the limited sensitivity of our AFM equipment, two etching methods were transferred to 10 *μ*m trenches. To guarantee reliable roughness data for them, the etching depth of the 10 *μ*m trenches was controlled to 120 nm, identical to that of the sub-100 nm trenches. As shown in Figure 2c,d, the RMS value of the trench etched by WA-SE is 2.475 nm. In contrast, that etched by WOA-SE is only 0.248 nm. It is 1/10 of the former. Obviously, the bombardment effect of Ar will damage the surface, increasing its roughness. In this regard, the absence of Ar during the SE process is more beneficial to the surface.

As shown in Figure 2e, the field-effect mobilities (*μ*_field_) of the two schemes were extracted through capacitance-voltage (CV) measurements [24]. The peak mobility of the WA-SE is 1190.2 cm^2^/(V·s), which is much lower than the WOA-SE of 1421.3 cm^2^/(V·s). The difference in mobility is attributed to the large damage of Ar-containing etching, which enhances interface roughness scattering [25]. This causes certain damage to the barrier, leading to a further reduction in device mobility [26]. Therefore, removing Ar during the SE process is better. It should be clarified that what we measured in this work is the field-effect mobility, not the intrinsic mobility of the material. The field-effect mobility is usually lower than the intrinsic mobility due to additional scattering at the dielectric/semiconductor interface [27].

### 2.3. The Further Investigation of WOA-SE

**Figure 3:**
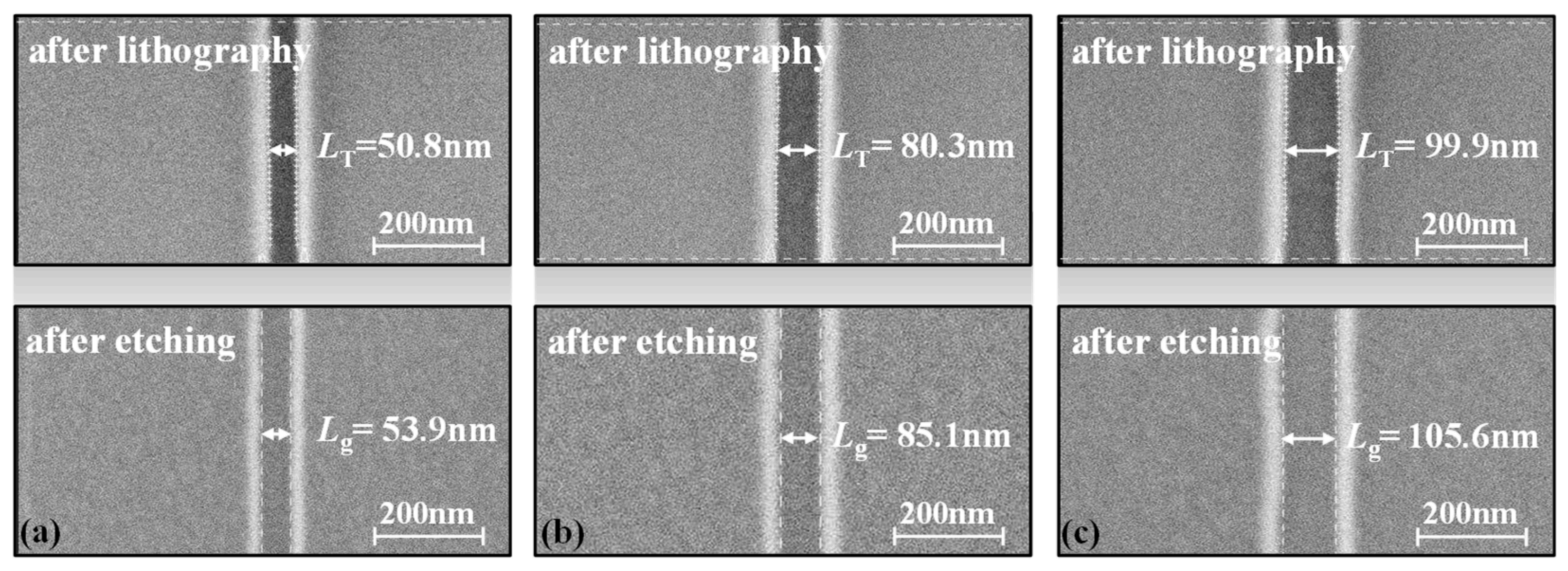
The CD-SEM images of (**a**) 50 nm, (**b**) 80 nm, and (**c**) 100 nm trenches after lithography and WOA-SE process.

To further investigate the features of the WOA-SE process, the different trenches were patterned by electron beam lithography (EBL), and then the WOA-SE process was followed. As shown in Figure 3a–c, they were measured using a Critical Dimension Scanning Electron Microscope (CD-SEM). For trenches of different dimensions, their *L*_E_ values are not identical. To investigate the universality of WOA-SE, we used EBL to fabricate trenches ranging from 50 nm to 1 *μ*m. Considering the actual *L*_g_ of current RF HEMTs [28,29], most of the lithographic trench lengths (*L*_litho_) are below 200 nm, with fewer large-size trenches. After etching, the dimensions of various trenches were measured by CD-SEM. Then we calculated the value of *L*_E_ using the following equation:

*L*_E_ = *L*_g_ − *L*_litho_ (4)

**Figure 4:**
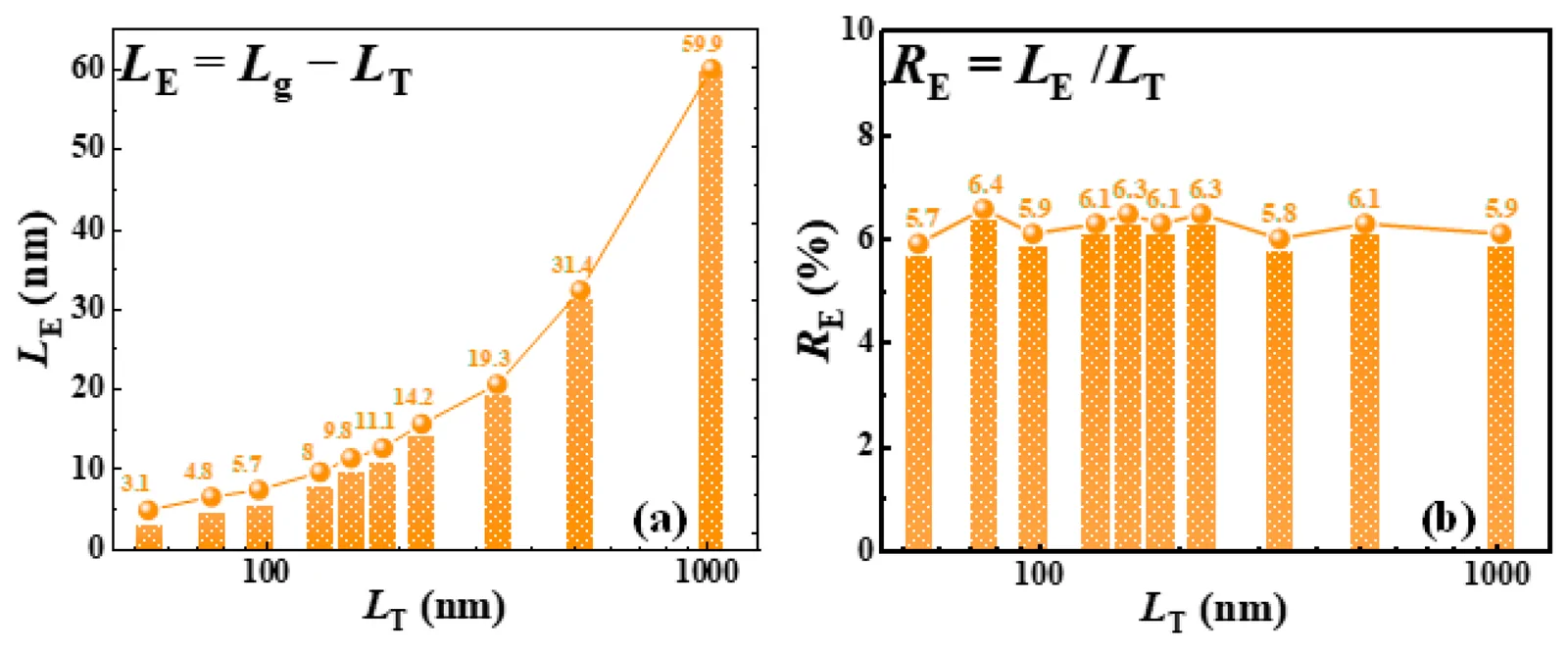
(**a**) *L*_E_ and (**b**) *R*_E_ of trenches ranging from 50 nm to 1 *μ*m.

As shown in Figure 4a, the *L*_E_ rises with increasing *L*_litho_. To investigate the other characteristics of WOA-SE, the expansion ratio (*R*_E_) of the lithographic trench was calculated. The corresponding formula can be expressed as follows:

*R*_E_ = *L*_E_/*L*_litho_ (5)

As shown in Figure 4b, the WOA-SE exhibits a consistent *R*_E_ for trenches of different sizes. The *L*_E_ can be predicted before etching, which is instructive for etching.

**Figure 5:**
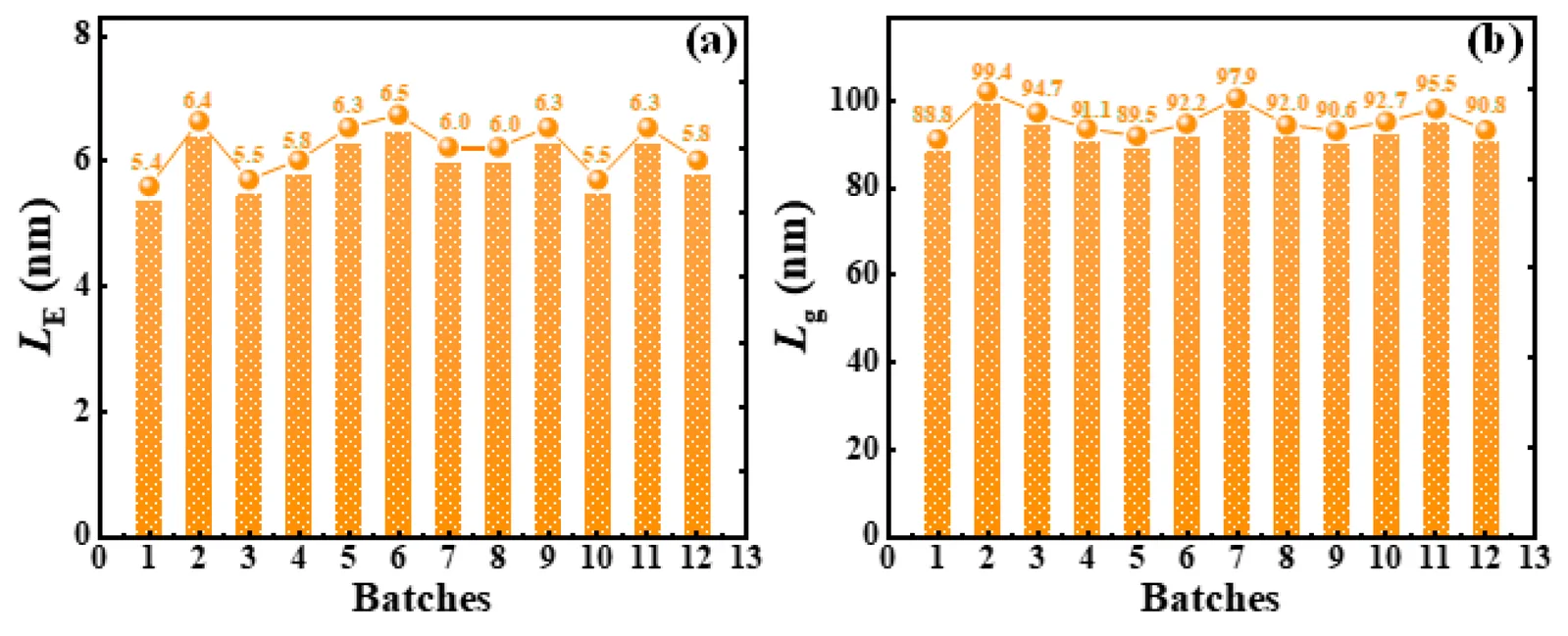
The statistical distribution of (**a**) *L*_E_ and (**b**) *L*_g_ across multi-batch experiments of the WOA-SE process.

The process robustness is critical. For the same etching process, first, wafer-to-wafer variations can cause inconsistent etching results. To investigate this effect, six different wafers were adopted for verification experiments. Second, intra-wafer non-uniformity leads to etching deviations at different positions on a single wafer. Therefore, comparative experiments were performed on different regions of each wafer, forming 12 test groups. As shown in Figure 5a,b, the results demonstrate that the *L*_E_ and *L*_g_ exhibit favorable consistency across different wafers and locations. This proves that the WOA-SE process has good reproducibility.

### 2.4. Device Fabrication

**Figure 6:**
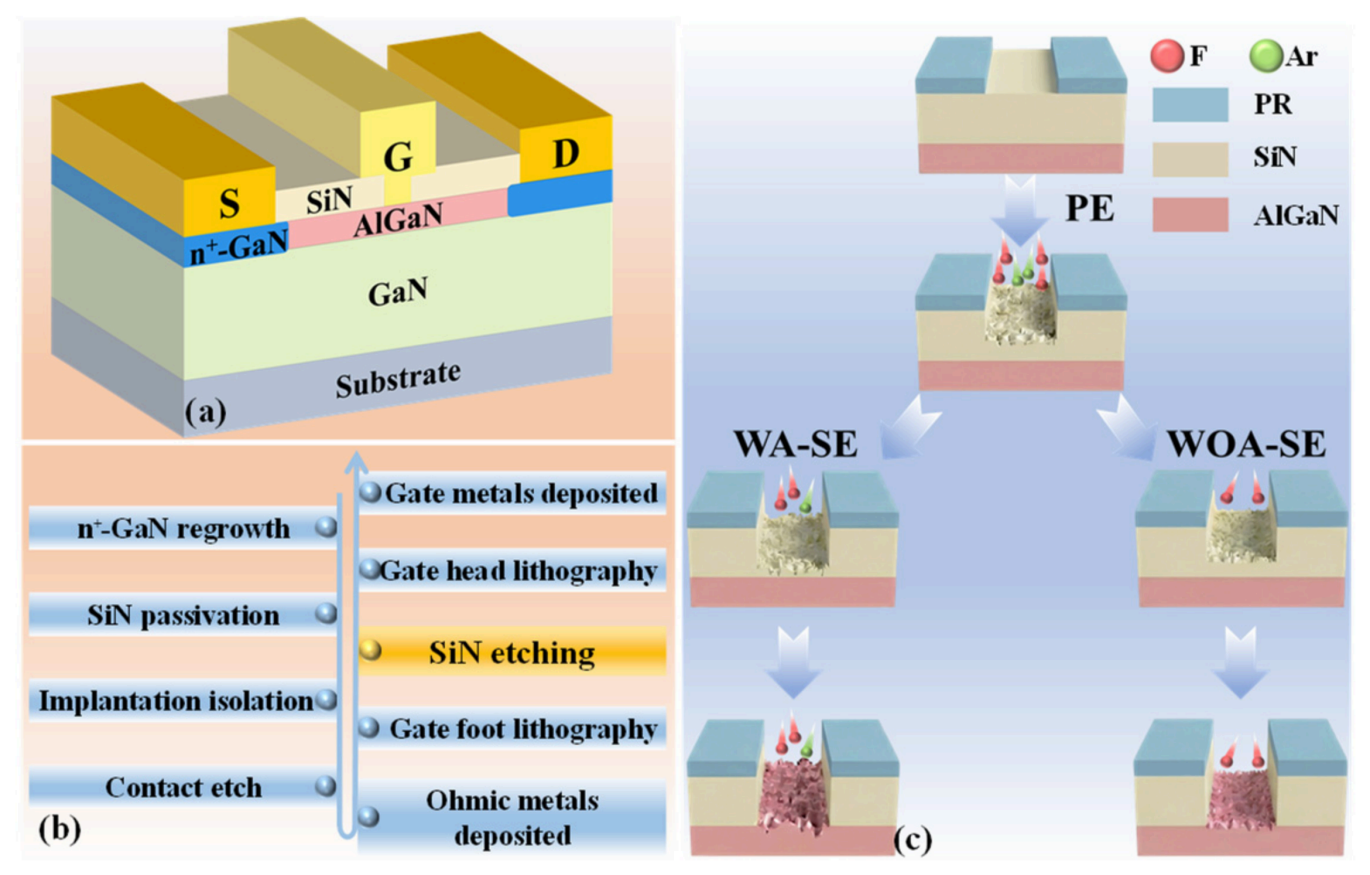
(**a**) Schematic of the cross section of the AlGaN/GaN HEMTs. (**b**) The fabrication process of the scaled-*L*_g_ device. (**c**) Schematic illustration of two different etching methods.

The cross-structure of the thin-barrier AlGaN/GaN HEMT is shown in Figure 6a. The epitaxial material was grown on the SiC substrate by metal-organic chemical vapor deposition (MOCVD) consisting of a GaN buffer and channel layer and a 12 nm Al_0.25_Ga_0.75_N barrier layer from bottom to top. Figure 6b shows the fabrication process of the scaled-*L*_g_ device, where the SiN etching process is the crucial step. During the device fabrication process, Cl-based etching was performed on AlGaN and GaN in the ohmic contact region, followed by the regrowth of an n^+^-GaN layer. Subsequently, 120 nm SiN was deposited via plasma-enhanced chemical vapor deposition (PECVD). After implantation isolation and contact etch, ohmic metals were deposited using the electron beam evaporation technique. Through the transmission line model (TLM) test, we measured ohmic contact resistance (*R*_c_) to be 0.121 Ω·mm. Afterwards, the gate foot region was formed by the EBL, followed by the removal of SiN using F-based dry etching. Figure 6c shows the processes of two different etching methods. After etching, a sub-100 nm gate foot was formed. Subsequently, the 400 nm gate head structure was defined using the stepper lithography technology. Finally, the gate metal was deposited via the electron beam evaporation technique. For the fabricated AlGaN/GaN HEMT, the source-drain spacing (*L*_SD_) is 2 *μ*m, and the 400 nm T-gate is located at the center between the source and drain regions.

### 2.5. Characteristics Comparison of the Two Devices

**Figure 7:**
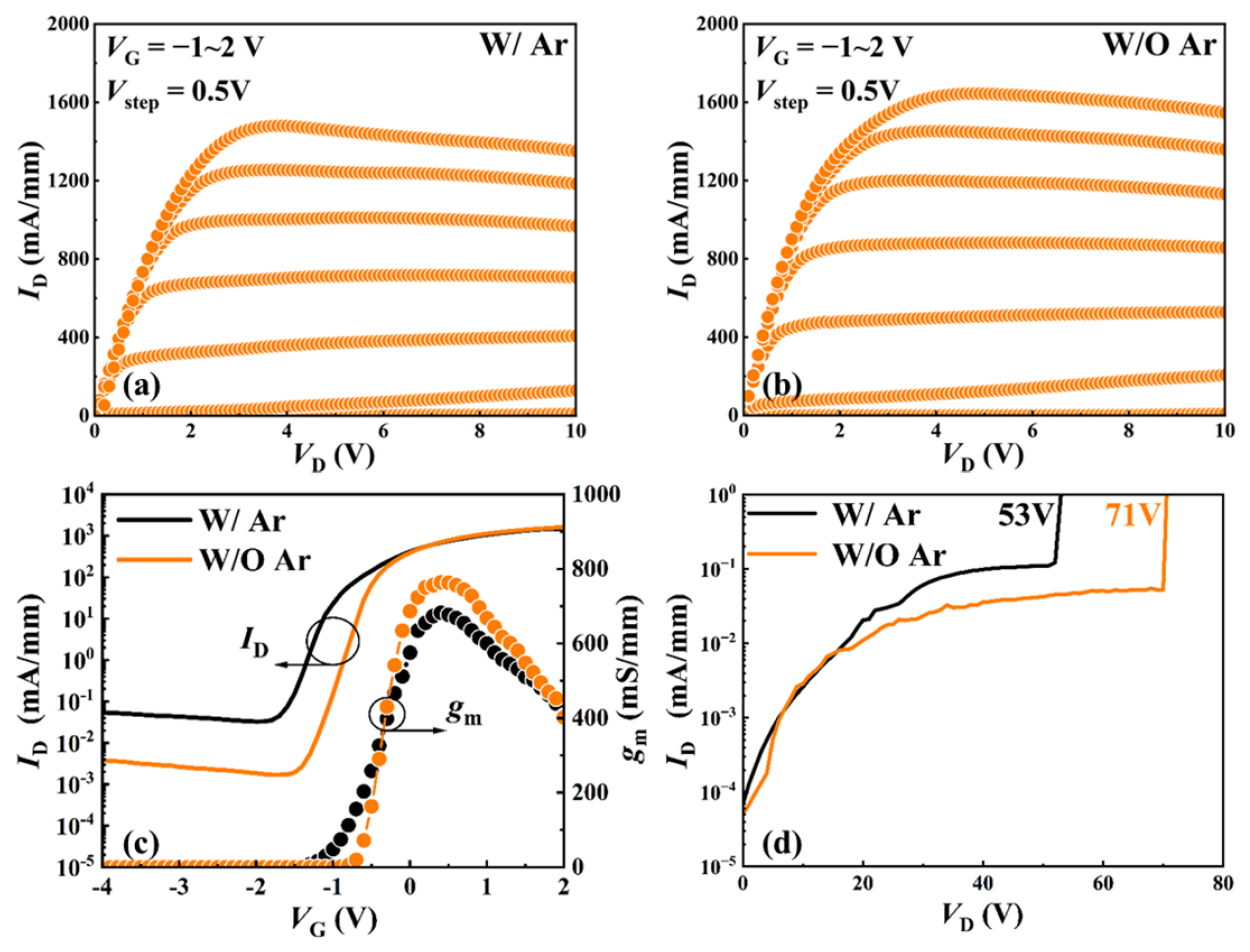
Output characteristics of (**a**) WA-HEMT and (**b**) WOA-HEMT. (**c**) Transfer characteristics on a logarithmic scale of two devices. (**d**) The *V*_BK_ of two devices.

WA-HEMT and WOA-HEMT are fabricated with WA-SE and WOA-SE technologies. Figure 7a,b shows their output characteristics. At gate voltage (*V*_G_) = 2 V, the maximum drain current density (*I*_DS.max_) can reach 1479.9 mA/mm (WA-HEMT) and 1644.2 mA/mm (WOA-HEMT). Figure 7c shows a peak transconductance (*g*_m.max_) of 682.5 mS/mm (WA-HEMT) and 764.1 mS/mm (WOA-HEMT), respectively. The threshold voltage (*V*th) is −1.3 V (WA-HEMT) and −0.8 V (WOA-HEMT), which is defined as the *V*_G_ at *I*_DS_ reaches 1 mA/mm. It can also be seen that the leakage current in the off-state is 5 × 10^−2^ mA/mm (WA- HEMT) and 3 × 10^−3^ mA/mm (WOA-HEMT). The minimum subthreshold swing (*SS*) values are 187.4 mV/dec (WA-HEMT) and 170.9 mV/dec (WOA-HEMT). These differences indirectly indicate that the WOA-SE process introduces fewer interfacial traps. As shown in Figure 7d, the breakdown voltage (*V*_BK_) reached 53 V (WA-HEMT) and 71 V (WOA-HEMT), with an improvement of 25%. The WOA-HEMT exhibits more prominent DC characteristics. This indicates that Ar-free etching can reduce etching damage [17].

**Figure 8:**
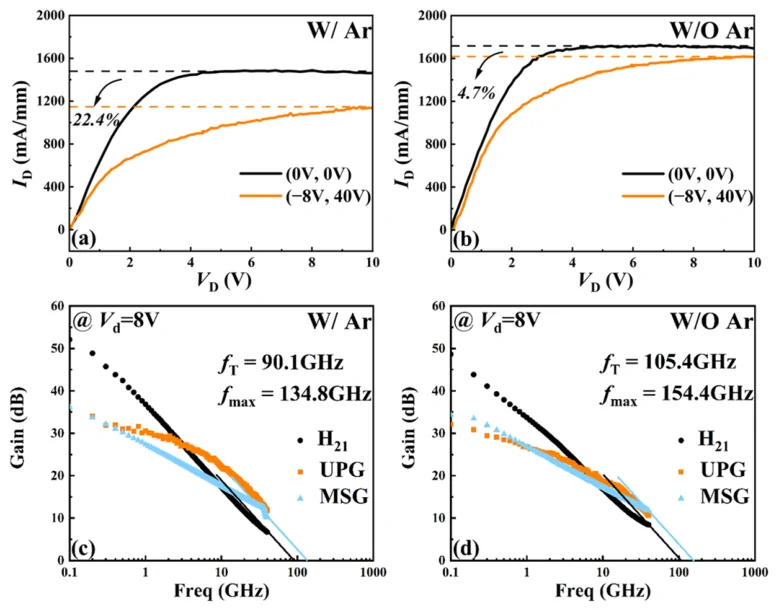
Current collapse characteristics of (**a**) WA-HEMT and (**b**) WOA-HEMT. Small-signal characteristics of the (**c**) WA-HEMT and (**d**) WOA-HEMT.

Figure 8a,b shows the current collapse characteristics. Quiescent bias conditions were biased at (*V*_GSQ_, *V*_DSQ_ = 0 V, 0 V) and (*V*_GSQ_, *V*_DSQ_ = −8 V, 40 V). The pulse width is 500 ns with a duty cycle of 1/10. The current collapse percentages of the WA-HEMT and the WOA-HEMT were 22.4% and 4.7%, respectively. This indicates that the WOA-HEMT exhibits more excellent stability. Figure 8c,d shows the small-signal characteristics. At drain-source voltage (*V*_DS_) = 8 V, the WA-HEMT achieved a gain cutoff frequency (*f*_T_) of 90.1 GHz and a maximum oscillation frequency (*f*_MAX_) of 134.8 GHz. For WOA-HEMT, its *f*_T_/*f*_MAX_ exhibited 105.4/154.4 GHz, which are both better than WA-HEMT. Ar-free etching results in low surface roughness, which reduces carrier scattering and improves carrier mobility, thereby increasing the *MSG* and *f*_MAX_ of the device.

**Figure 9:**
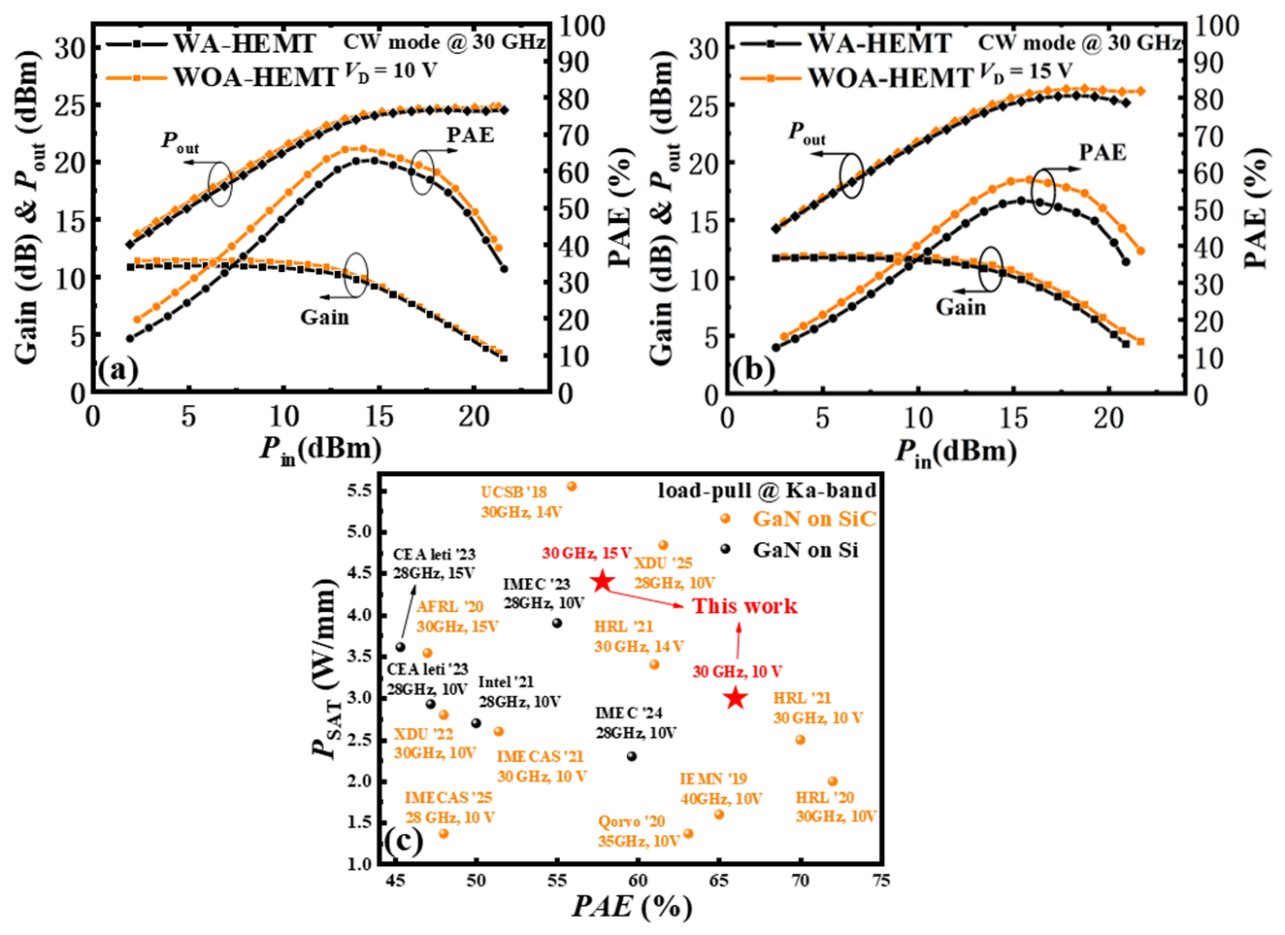
CW power performances at 30 GHz of WA-HEMT and WOA-HEMT at *V*_DSQ_ = (**a**) 10 V and (**b**) 15 V. (**c**) Comparison of *P*_SAT_ and *PAE* between GaN HEMTs for Ka-band wireless communication applications.

An on-wafer load-pull system at *f* = 30 GHz was used to evaluate the continuous wave (CW) power performance. Both the source and the load impedance were tuned to optimize the *PAE*. As shown in Figure 9a, the WA-HEMT exhibited a *P*_sat_ of 2.8 W/mm and a *PAE*_max_ of 62.7% at *V*_DSQ_ of 10 V. In contrast, the WOA-HEMT exhibited a *P*_sat_ of 3.0 W/mm and a peak *PAE* of 66.0%, with improvements of 7.1% and 5.3%, respectively. As shown in Figure 9b, with *V*_DSQ_ of 15 V, WA-HEMT exhibited a *P*_sat_ of 3.8 W/mm and a *PAE*_max_ of 52.1%, and WOA-HEMT achieved 4.4 W/mm and 57.8%, increased by 15.7% and 10.7%, respectively. This is attributed to the higher *MSG* and *f*_MAX_ of the WOA-HEMT, which can improve the signal amplification capability and power conversion efficiency. The *P*_sat_ and *PAE*_max_ results of the Ka-band GaN-based devices, as reported in references [6,30,31,32,33,34,35,36,37,38,39,40,41], are summarized in Figure 9c. Compared with other GaN-on-Si HEMT and GaN-on-SiC HEMT technologies, the WOA-HEMT demonstrates advantages in both *PAE*_max_ and *P*_sat_, which are attributed to the Ar-free low-damage etching technology.

## 3. Conclusions

The proposed high-anisotropy and low-damage etching method enables stable fabrication of sub-100 nm *L*_g_. Its core lies in achieving Ar-free etching during the SE process. Furthermore, this method significantly reduces the roughness of the thin AlGaN barrier layer, thereby suppressing mobility degradation. The fabricated AlGaN/GaN HEMT exhibited excellent characteristics, with *I*_DS.max_ of 1644.2 mA/mm and *g*_m.max_ of 764.1 mS/mm. At (*V*_GSQ_, *V*_DSQ_ = −8 V, 40 V), the current collapse was only 4.7%. At *V*_DS_ of 8 V, the *f*_T_/*f*_MAX_ values were 105.4/154.4 GHz, respectively. At 30 GHz, the device achieved a *P*_sat_ of 4.4 W/mm and a *PAE* of 66.0%, with significant improvements of 15.7% and 5.3%, respectively, compared to the device fabricated with Ar etching. These results demonstrate that the AlGaN/GaN HEMT based on the Ar-free low-damage etching technology holds considerable potential for RF PA applications.

## Data Availability

The original contributions presented in this study are included in the article. Further inquiries can be directed to the corresponding author.
